# *Mycobacterium avium* lysate induces matrix metalloproteinase-1 in intestinal tissue and peripheral blood: Observations from selected hospital based Zambian adults

**DOI:** 10.1016/j.ijid.2018.03.020

**Published:** 2018-06

**Authors:** Gershom Chongwe, Charles Michelo, Edford Sinkala, Violet Kayamba, Jean-Baptiste Nzayisenga, Francis Drobniewski, Paul Kelly

**Affiliations:** a Department of Epidemiology & Biostatistics, University of Zambia School of Public Health, Lusaka, Zambia; b University of Zambia, Strategic Centre for Health Systems Metrics & Evaluations (SCHEME), School of Public Health, Lusaka, Zambia; c Department of Internal Medicine, University of Zambia School of Medicine, Lusaka, Zambia; d Department of Surgery, University Teaching Hospital, Lusaka, Zambia; e Infectious Diseases and Immunity, Department of Medicine, Imperial College, London, UK; f Blizard Institute, Barts & The London School of Medicine, Queen Mary University of London, London, UK

**Keywords:** Matrix Metalloproteinases, Cytokines, Non-tuberculous mycobacteria, Zambia, Environmental enteropathy

## Abstract

**Objectives:**

Environmental enteropathy is prevalent in low-income countries, although its aetiology is unknown. We investigated if *Mycobacterium avium* antigens, which are commonly found in the environment, could contribute to its pathogenesis in a population known to have widespread environmental enteropathy.

**Methods:**

Routine endoscopy patients at the University Teaching Hospital, Lusaka whose endoscopy results were normal submitted duodenal biopsies and whole blood samples. Samples were stimulated with *M. avium* lysate over 24 h while unstimulated samples served as negative controls. Matrix metalloproteinase (MMP) and cytokine response in supernatants were quantified using ELISA and cytometric bead array.

**Results:**

Samples from 48 patients (56% women) were analysed, with a median age of 35 years (IQR 27.5, 50.5). *M. avium* induced the secretion of a wide-range of Th1, Th2 and Th17 cytokines in blood but only IL-1β and IL-6 in duodenal tissue. However it differentially induced the secretion of MMP-1 in duodenal tissue compared to negative controls (p = 0.004). A similar MMP-1 response but with lower concentrations was observed in blood.

**Conclusion:**

The induction of MMP-1 and cytokines by *M. avium* in duodenal tissue suggests that environmental mycobacteria could contribute to the epithelial disruption seen in environmental enteropathy, and a need to further explore possible biomarkers that may predict this exposure in at-risk populations.

## Highlights

• *Mycobacterium avium* could remodel the mucosa and lead to environmental enteropathy.
• *M. avium* induced the secretion of MMP-1 in duodenal tissue and whole blood.
• *M. avium* also induced the secretion of IL-1β and IL-6 in duodenal tissue.

## Background

Environmental enteropathy is a widespread subclinical disturbance found in low-income countries in individuals exposed over time to poor sanitation and hygiene. It has been identified as a possible cause of stunting and malnutrition, oral vaccine failure and impaired development in children from low-income countries. Although much about its cause remains unknown, it has been proposed to result from increased T-cell activation arising from the repeated exposure to insanitary conditions in many poor regions of the world (Korpe and Petri, 2012, Veitch et al., 2001). Several pathways have been proposed, all leading to specific morphological and functional derangements involving intestinal mucosal disruption and increased susceptibility to infection (Naylor et al., 2015, Prendergast and Kelly, 2012).

Matrix metalloproteinases (MMPs) are a group of zinc dependent enzymes which degrade structural proteins. Specifically it has been documented that MMP-1 and −8 degrade many types of collagen as well as proteoglycans, while MMP-2 and −9 degrade gelatin, type IV collagen, fibronectin and elastins (Lee, 2014, Ravi et al., 2007). They have been implicated in the pathogenesis of inflammatory bowel disease due to their role in tissue resorption and re-modelling of the extracellular matrix (O’Sullivan et al., 2015, Ravi et al., 2007). It has been suggested that the degradation of both extracellular and intracellular structural molecules by MMPs can contribute to impairment of the intestinal epithelial barrier seen in inflammatory bowel disease. MMPs are activated by growth factors, hormones, various cytokines, and cellular transformation (de Bruyn et al., 2016).

Nontuberculous mycobacteria are common environmental pathogens that are frequently being found to be responsible for disease in humans (Falkinham, 2002, Mirsaeidi et al., 2013). Given the ubiquity of NTM, their constant passage through the gut (Chongwe et al., 2017) and their ability to induce various cytokines through T-cell activation, notwithstanding that the exact mechanism for enteropathy is still unknown, we hypothesised that these NTMs could play a role in the pathogenesis. We set out to investigate the role of *Mycobacterium avium* in inducing inflammatory cytokine secretion as well as the secretion of MMPs in human gut biopsies in a population known to have widespread environmental enteropathy (Kelly et al., 2016, Kelly et al., 2004).

## Methods

### Patients and recruitment

We recruited participants from among consenting adults 18 years and older reporting for routine endoscopy at the University Teaching Hospital, Lusaka between July and December 2014. Selection of participants was based on a simple random sampling from a list of booked patients on each clinic day. A structured questionnaire was used to extract baseline data and to collect information on possible risk factors for tuberculosis or nontuberculous mycobacterial infection. This included collection of information on HIV status as well as having a previous Bacillus Calmette–Guerin (BCG), vaccination (verified by a scar on the arm) and self-reported history of TB.

### Biopsy collection and processing

Diagnostic endoscopy procedures were performed using Pentax EG2990i endoscopes (Pentax, Tokyo, Japan). During endoscopy three biopsies were taken from the second part of the duodenum, in those patients in whom the endoscopy was found to be normal, and immediately placed into a 2 ml cryovial filled with 1 ml culture media composed of five volumes Dulbecco’s modified Eagle’s medium, five volumes National Cancer Tissue Culture-135 medium and one volume of newborn calf serum, all from Sigma Aldrich, (Dorset, UK). The biopsies were cultured with *M. avium* lysate in tissue culture medium within two hours in an environment with 95% O_2_/5% CO_2_ at 37 °C for 24 h as previously described (Dhaliwal et al., 2003, Dhaliwal et al., 2009). For each participant, the first biopsy was placed on a centre well culture dish (Sigma Aldrich, Dorset, UK) with no stimulant added and this served as a negative control, the second biopsy was stimulated with 10 μl of *M. avium* lysate while the third biopsy was stimulated with 10 μl Staphylococcus enterotoxin B (SEB) antigen. An additional positive control to confirm the validity of the experimental model using *Salmonella typhimurium* lipopolysaccharide (LPS) was also used (results not shown).

### Assays for MMPs and cytokines

After 24 h of incubation, the supernatant was collected and stored at −80 °C. To quantify cytokines, the human cytometric bead array Th1/Th2/Th17A kit (Becton, Dickinson and Company, San Jose, California, USA) was used to detect interleukin 2 (IL-2), interleukin 4 (IL-4), interleukin 6 (IL-6), interleukin 10 (IL-10), interleukin 17A (IL-17A), Tumour Necrosis Factor alpha (TNF-α) and Interferon Gamma (IFN-γ). This was performed on a BD FACSVerse flow cytometer, and analysed using BD FCAP array software. Measurement of IL-1β and IL-12 were performed by ELISA as they are not included in the CBA kit. MMP-1, -2, -8, and -9 were also quantified using ELISA assays. Information was also collected on history of tuberculosis (TB) and having a BCG scar, while HIV testing was done on all participants. Cell viability (results not shown) was assessed using the MTT-based in vitro toxicology assay kit (Sigma, Dorset, UK) which confirmed viability of the tissue during incubation. The results were exported into Stata 14 software (College Station, Texas, USA) for analysis. A subset of 13 samples chosen at random were used to quantify levels of MMP-1, -2, -8 and -9 secretion using ELISA methods (R&D Systems, Abingdon, UK).

### Analysis

MMP or cytokine quantities were expressed in nanograms per ml for MMPs and picograms per ml for other cytokines and this data was entered in Excel and analysed using Stata version 14 (Stata Corp., College Station, Texas). The Shapiro–Wilk test for normality showed that these data were non-normally distributed and so the Wilcoxon signed rank test was used to compare cytokines or MMP concentrations in supernatants from *M. avium* stimulated samples and unstimulated control samples from the same individuals. Spearman’s correlation was used to check for association between and among the different cytokines and MMPs. Secretion of cytokines or MMPs was stratified by HIV status, having a previous Bacillus Calmette–Guerin (BCG) vaccination (verified by a scar on the arm) and self-reported history of TB. The difference in cytokine secretion between each *M. avium*-stimulated sample and their negative control was estimated using panel random effects multivariable linear regression analysis with maximum likelihood estimation (xtreg in Stata), using an investigator-led backward regression. Akaike and Bayesian information criteria were used for model selection, and a p-value of less than 0.05 was considered significant. Positive controls were included in all experiments but used only to confirm the viability of each experiment and were not included in pairwise comparisons. The study was approved by the University of Zambia Biomedical Research Ethics Committee (Reference # 015-07-12).

## Results

Overall (n = 48) we recruited 21 men and 27 women whose median age was 35 (IQR 27.5, 50.5) years; women were slightly older than men (Table 1). Six (13%) of the participants were HIV positive. Most of the participants (74%) had evidence of previous BCG vaccination, while 15% had a previous history of TB.

**Table 1:** Characteristics of participants recruited for the in-vitro study to determine the secretion of cytokines and MMPs in duodenal samples from 48 individuals.

Variable	Overall [n (%)]	Female [n (%)]	Male [n (%)]
Age^*^	35.0 (27.5, 50.5)^*^	35.5 (26, 52.5)^*^	35.0 (29.5, 41.0)^*^
Sex [n (%)]	48	27 (54)	21 (46)

BCG scar			
Present	23	10 (44)	13 (56)

History of TB			
Present	7	3 (43)	4 (57)
HIV status			
Seropositive	6	4 (67)	2 (33)

Presenting symptoms			
Abdominal pain	39 (81)	22 (56)	17 (44)
Vomiting	8 (17)	5 (62)	3 (38)
Diarrhoea	7 (15)	3 (43)	4 (57)
Night sweats	4 (8.3)	1 (25)	3 (75)
Cough	3 (7.4)	2 (67)	1 (33)

*Note*: *Age is expressed in median (Interquartile Range, IQR).

The most common presenting symptom was abdominal pain, which was the indication for endoscopy in 39 (82%) patients. Other symptoms present at the time of endoscopy were vomiting (17%), diarrhoea (15%) and cough (7.4%) as shown in Table 1. The majority of tissue, therefore, came from patients with functional dyspepsia.

### *M. avium* induces IL-1β and IL-6 secretion in duodenal biopsies

**Figure 1:**
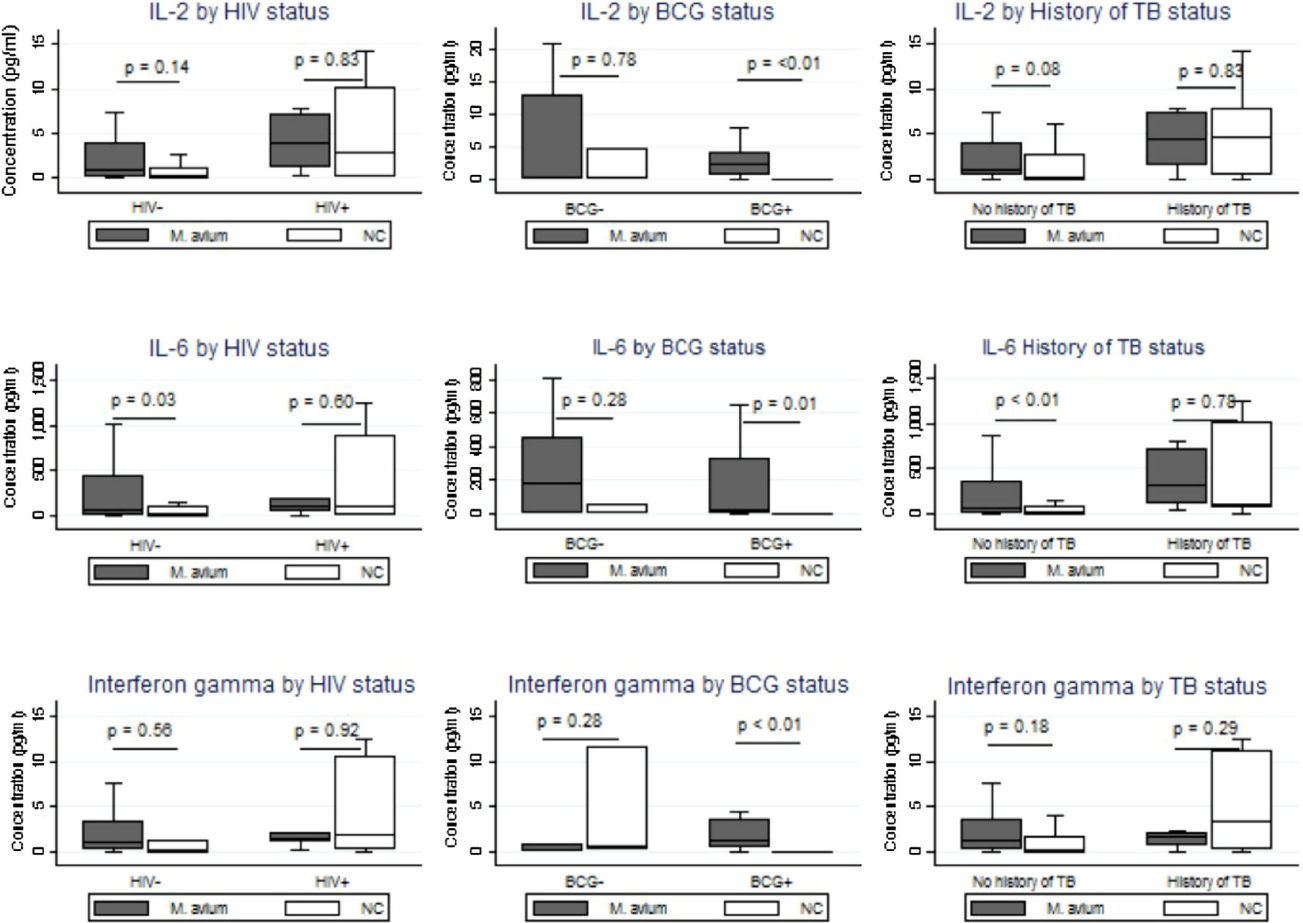
Secretion of some gut inflammatory cytokines among endoscopy patients in Lusaka, stratified by HIV, BCG and history of TB. *Notes*: p values comparing *Mycobacterium avium* versus negative controls (NC) calculated using the Wilcoxon signed rank test as sets of biopsies were collected from the same individuals.

Interleukin 1β and IL-6 were significantly higher (p = 0.002 and 0.04 respectively) in supernatants from duodenal biopsies from the same individuals cultured with *M. avium* than from controls (Table 2). The secretion of IL-6 in *M. avium* stimulated samples was higher in patients with a history of TB than those without a history of TB (Figure 1). Intestinal tissue from HIV negative participants secreted more IL-6 but, not in any other cytokines. Induction of IL-2, IL-6, IL-10 (not shown), IL-17A, IFN-g and was higher in participants with BCG scars, but only IL-6 were induced more in participants with no past history of tuberculosis (Figure 1). Stratifying the IL-1, IL4 and IL-12 by HIV status, having a BCG scar or history of TB did not show any differences in our patients. Similarly, the secretion of the cytokines did not show any differences when stratified by age group and sex.

**Table 2:** Secretion of cytokines by duodenal explants into supernatant during stimulation with *Mycobacterium avium* lysate.

Cytokine^*^	Number	Amount secreted after stimulation with *M. avium* (pg/ml)^a^	Amount secreted by the negative control (pg/ml)^a^	P value^b^
Interleukin 1β	13	23.6 (10.0, 83.0)	0.14 (0.00, 1.36)	**0.002^§^**
Interleukin 2	30	2.18 (0.34, 4.32)	0.26 (0.00, 4.66)	0.25
Interleukin 4	30	0.18 (0.00, 0.70)	0.18 (0.00, 1.84)	0.31
Interleukin 6	30	77.1 (0.94, 408.4)	16.2 (0.00, 104.4)	**0.04**
Interleukin 10	30	0.08 (0.00, 0.49)	0.01 (0.00, 0.92)	0.36
Interleukin 12	13	7.84 (5.10, 15.8)	9.48 (4.29, 14.6)	0.97
Interleukin 17A	30	12.0 (2.68, 28.3)	1.15 (0.00, 19.6)	0.25
TNF – α	30	0.31 (0.00, 2.34)	0.24 (0.00, 2.71)	0.91
Interferon γ	30	1.27 (0.28, 3.16)	0.23 (0.00, 3.39)	0.57

*Notes*: ^a^All the figures are medians with interquartile range, in pg/ml. ^b^p-value calculated using the Wilcoxon signed-rank test. ^*^Except for IL-1Β and 12, for which ELISA was used, the other cytokines were quantified using flow cytometry via the cytometric bead array. §: Boldface figure signifies statistical significance.

In multivariable regression analysis of data from duodenal samples (Table 3), the results showed that *M. avium*-stimulated duodenal samples expressed 156 pg of IL-6 more than unstimulated samples (95% CI 7.6, 304; p = 0.04) after adjusting for the effect of history of TB, previous BCG vaccination, age and sex. We did not detect any differences in secretion of IL-17A, IL-10, IL-4, IL-2, IFN-γ and TNF-α in the gut between stimulated samples and unstimulated samples.

**Table 3:** Panel linear regression analyses showing the effect of *Mycobacterium avium* stimulation on secretion of each cytokine.

Cytokine	Unadjusted			Adjusted		
	Mean cytokine secretion (SD) [pg/ml]	Mean difference (95%CI)	p value^*^	Mean difference (95%CI)	p value^*^	Intra-cluster correlation coefficient (Rho)
IL-17A						
Not stimulated	19.3 (30.2)	Ref	0.14	Ref	0.07	–
Stimulated with *M. avium*	20.5 (24.5)	1.72 (−8.56, 12.0)		11.8 (−0.97, 24.7)^1^		

IL-10						
Not stimulated	0.60 (1.01)	Ref	0.64	Ref	0.52	0.33
Stimulated with *M. avium*	0.57 (1.24)	0.09 (−0.27, 0.44)		0.08 (−0.17, 0.35)^2^		

IL-6						
Not stimulated	202.0 (365.5)	Ref	0.36	Ref	**0.04^§^**	0.42
Stimulated with *M. avium*	271.7 (440.1)	74.6 (−84.0, 233.5)		155.8 (7.64, 304.0)^3^		

IL-4						
Not stimulated	1.31 (2.43)	Ref	0.46	Ref	0.60	0.02
Stimulated with *M. avium*	0.93 (1.70)	−0.26 (−0.93, 0.42)		−0.06 (−0.16, 0.28)^4^		

IL-2						
Not stimulated	3.46 (7.98)	Ref	0.82	Ref	0.36	0.75
Stimulated with *M. avium*	6.46 (23.6)	0.28 (−2.16, 2.71)		3.84 (−4.47, 12.1)^5^		

IFNG						
Not stimulated	3.52 (8.33)	Ref	0.74	Ref	0.45	0.64
Stimulated with *M. avium*	3.68 (9.02)	−0.41 (−2.80, 1.99)		1.41 (−2.23, 5006)^6^		

TNF						
Not stimulated	4.03 (10.9)	Ref	0.40	Ref	0.97	0.74
Stimulated with *M. avium*	2.53 (5.61)	−1.32 (−4.43, 1.79)		−0.01 (−0.39, 0.41)^7^		

*Notes*: ^*^p value was calculated using panel random effects linear regression with maximum likelihood estimation. §: Boldface figure signifies statistical significance. ICC: Intracluster Correlation Coefficient; each row represents a separate multiple linear regression model. 1: adjusted for BCG vaccination, age and history of TB; 2: adjusted for sex, age, history of TB, HIV and previous BCG vaccination; 3: adjusted for sex, age, history of TB and previous BCG vaccination; 4: adjusted for sex, age, history of TB, HIV and previous BCG vaccination; 5: adjusted for sex, age, history of TB, previous BCG vaccination; 6: adjusted for sex, age and BCG vaccination; 7: adjusted for sex and BCG vaccination.

### *M. avium* induces the secretion of Th1 and Th2 response but not Th17 response in whole blood

**Figure 2:**
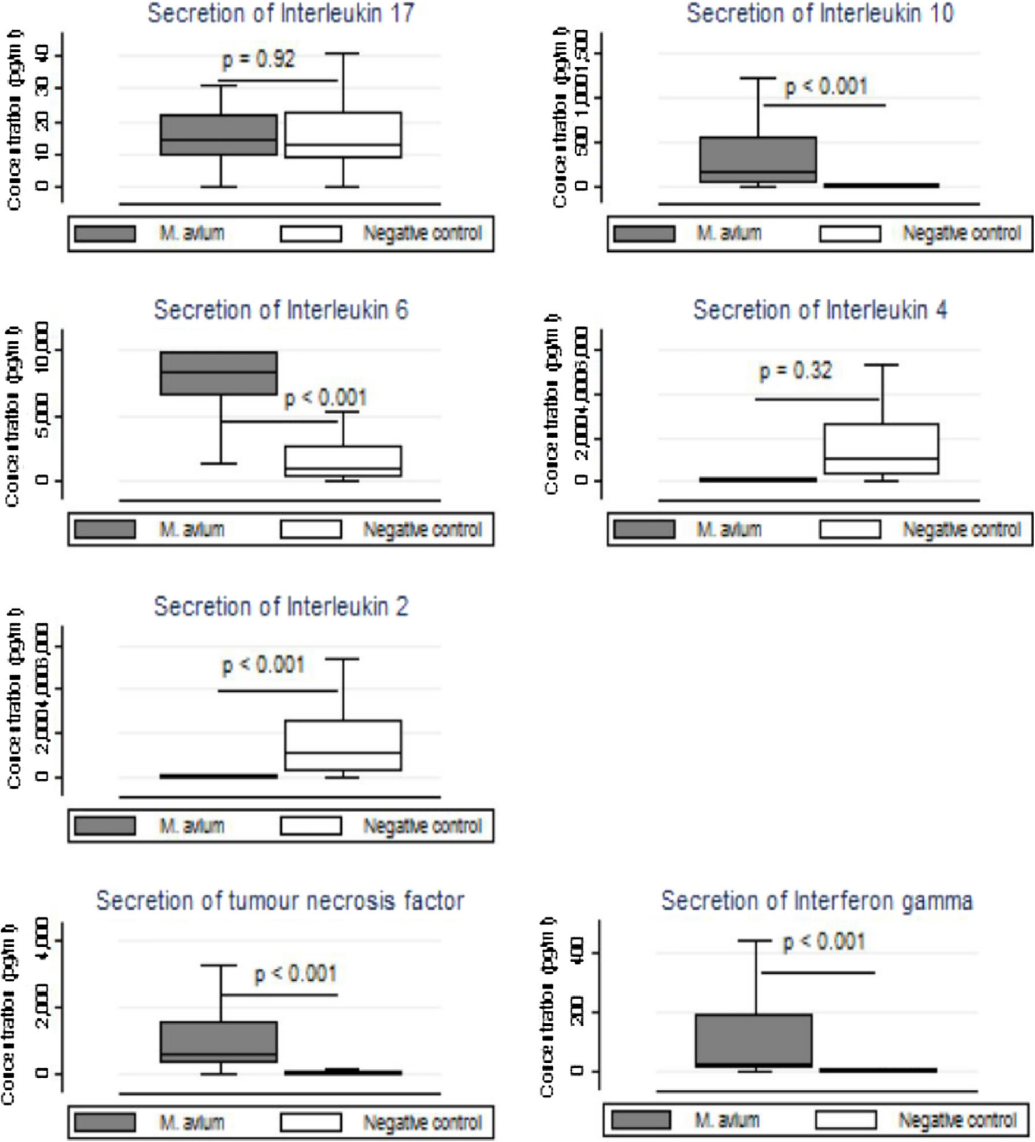
Secretion of cytokines in whole blood after stimulation with *Mycobacterium avium* complex. *Notes*: p values comparing *Mycobacterium avium* versus negative controls (NC) calculated using the Wilcoxon matched pairs signed rank test.

*M. avium* lysate stimulation of whole blood samples showed that *M. avium* induced the secretion of much higher levels of cytokines than those produced by duodenal tissue. Unlike in the gut, *M. avium* significantly increased whole blood secretion of the regulatory cytokine IL-10, (p < 0.001), and the pro-inflammatory cytokines IL-2, IL-6, TNF-α and IFN-γ were all significantly higher in stimulated samples compared to unstimulated samples (Figure 2). IL-17A was not induced by *M. avium* stimulation. Multiple linear regression (Table 5) showed that *M. avium* induced the secretion of Th1 and Th2 cytokines but not Th17 cytokines in whole blood in this experiment.

### MMPs

Having observed these changes in cytokine secretion in blood, and IL-6 in intestinal tissue, we went on to test the hypothesis that MMPs might be induced alongside them in response to *M. avium* lysate. From among the 48 participants, we took a non-sex differentiated random sub-sample of 14 participants and this was based on what could be handled by the ELISA kit. It turned out that there were five males and eight females in this sub-sample. The median age for this group was 32.5 years (IQR 27, 36), with males having a median age of 31.5 (IQR 27, 36) years and females with 34 (IQR 25, 40) years. They were therefore similar to the whole group.

The secretion of MMP-1 in supernatants from duodenal tissue stimulated with *M. avium* was increased compared to the negative controls, but secretion of MMP-2, -8 and -9 in *M. avium* stimulated tissues was not significantly increased (Table 4).

**Table 4:** Panel linear regression analysis showing the effect of *M. avium* stimulation on cytokine secretion in whole blood.

Cytokine	Unadjusted			Adjusted		
	Mean cytokine secretion (SD) [pg/ml]	Mean difference (95%CI)	p value^§^	Mean difference (95%CI)	P value	Intra-cluster correlation coefficient (rho)
IL-17A						
Not stimulated	17.2 (14.4)	Ref	0.63	Ref	0.91	0.002
Stimulated with *M. avium*	19.4 (24.1)	2.19 (−6.76, 11.2)		0.52 (−8.60, 9.64)^1^		

IL-10						
Not stimulated	9.59 (17.5)	Ref	**<0.01**^**§**^	Ref	**<0.01**	0.00
Stimulated with *M. avium*	434.2 (641.0)	422.3 (203.1, 641.6)		432.3 (213.3, 651.4)^2^		

IL-6						
Not stimulated	2028.6 (2666.8)	Ref	**<0.01**	Ref	**<0.01**	0.19
Stimulated with *M. avium*	7511.2 (2513.5)	5447.2 (4370.8, 6523.6)		5423.2 (4315.8, 6530.7)^3^		

IL-4						
Not stimulated	0.43 (1.22)	Ref	**0.05**	Ref	**0.04**	0.00
Stimulated with *M. avium*	1.58 (3.59)	1.17 (−0.01, 2.34)		1.27 (0.07, 2.46)^4^		

IL-2						
Not stimulated	11.1 (51.5)	Ref	**0.03**	Ref	**0.02**	0.00
Stimulated with *M. avium*	271.9 (669.9)	260.7 (31.45, 490.0)		277 (54.21, 500.0)^5^		

IFNG						
Not stimulated	5.26 (17.9)	Ref	**<0.01**	Ref	**<0.01**	0.00
Stimulated with *M. avium*	241.86 (424.3)	236.6 (91.65, 381.5)		216.5 (82.64, 350.3)^6^		

TNF						
Not stimulated	197.0 (594.6)	Ref	**<0.01**	Ref	**<0.01**	0.42
Stimulated with *M. avium*	1366.9 (1673.8)	1098.2 (664.0, 1532.4)		1083.2 (634.2, 1532.3)^7^		

*Notes*: ^§^p value was calculated using panel random effects linear regression with the maximum likelihood estimation. §: Boldface figure signifies statistical significance. Each row represents a separate multiple linear regression model. 1: adjusted for history of TB and sex; 2: adjusted for sex, age, history of TB, HIV and previous BCG vaccination; 3: history of TB; 4: adjusted for sex, history of TB and previous BCG vaccination; 5: adjusted for sex and history of TB; 6: adjusted for sex, and History of TB; 7: adjusted for sex, age, history of TB, HIV and previous BCG vaccination.

**Table 5:** Secretion of MMPs in duodenal tissue in response to *M. avium* stimulation.

Cytokine	Stimulated with *M. avium* (ng/ml)^a^	Negative control (ng/ml)^a^	p value^b^
MMP-1	1.23 (0.02, 3.95)	0.04 (0.01, 0.97)	0.002
MMP-8	0.23 (0.00, 0.49)	0.00 (0.00, 0.23)	0.06
MMP 2	0.36 (0.07, 0.85)	0.23 (0.02, 0.26)	0.12
MMP-9	0.98 (0.00, 2.25)	0.56 (0.00, 1.38)	0.50

*Notes*: n = 13. ^a^All the figures are medians with interquartile range, in ng/ml. ^b^p-value calculated using the Wilcoxon signed-rank test.

**Figure 3:**
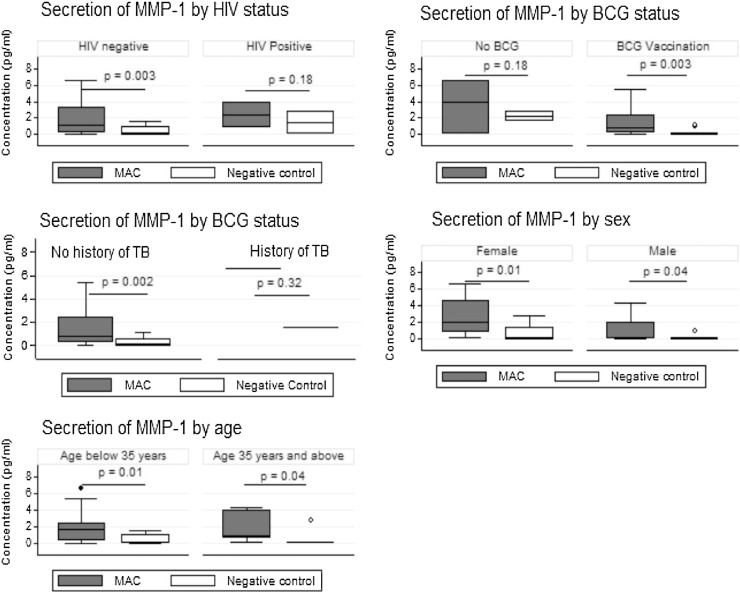
Stratified analysis of MMP-1 against HIV, BCG vaccination status, sex and age.

We analysed the changes in secretion of all the MMPs according to HIV status, having a BCG scar, gender and age group. The induction of MMP-1 was greatest in those who were HIV negative, in those with BCG vaccination scars, in younger participants and in women (Figure 3). However stratified analysis for MMP-2, -8 and -9 did not show any differences.

The secretion of MMP-1 in whole blood stimulated with *M. avium* was significantly higher than in unstimulated blood, p = 0.02. However, the level of MMP-1 expressed in blood was lower than that expressed by duodenal samples. When the subgroups HIV, previous BCG vaccination and history of TB were considered, the patterns of secretion of MMP-1 in the blood was similar to that expressed by duodenal samples.

### Correlation analysis

Using Spearman’s correlation analysis, duodenal MMP-1 secretion was positively correlated with that of IL-6 (*ρ* = 0.70, p = 0.01), while duodenal IL-17A secretion was correlated with that of IL-10, IL-6, IL-4, IL-2 interferon gamma and TNF (Table 6). Interleukin 10 secretion was correlated with the secretion of IL-4 and that of IL-2 (Table 6).

**Table 6:** Spearman’s correlation Matrix showing the association among Cytokines and Matrix Metalloproteinases in intestinal tissue.

	IL-17A												
IL-17A	1												
		IL-12											
IL-12	–	1											
			IL-10										
IL-10	0.57	–	1										
				IL-6									
IL-6	0.52	–	0.33	1									
					IL-4								
IL-4	0.58	–	0.56	–	1								
						IL-2							
IL-2	0.58	–	0.54	0.47	0.48	1							
							IL-1β						
IL-1β	–	–	–	–	–	–	1						
								INF-γ					
INF-γ	0.66	–	0.54	0.42	0.58	0.80	–	1					
									TNF-α				
TNF- α	0.51	–	0.50	–	0.74	0.39	–	0.56	1				
										MMP-1			
MMP-1	–	–	–	0.70	–	–	–	–	–	1			
											MMP-2		
MMP-2	–	–	–	–	–	–	–	–	–		1		
												MMP-8	
MMP-8	–	0.61	–	–	–	–	0.59	–	–	0.60	–	1	
													MMP-9
MMP-9	–	–	–	0.73	–	–	–	–	–	0.90	–	–	1

*Note*: the matrix is showing only correlations that were statistically significant.

## Discussion

Our results have shown that a lysate of *Mycobacterium avium* bacteria induced the secretion of IL-1β and MMP-1 in duodenal biopsies of healthy patients undergoing endoscopy. MMPs have long been implicated in the pathogenesis of inflammatory bowel disease, as well as other diseases that are characterized by destruction of the extracellular tissue such as rheumatoid arthritis, periodontal disease (Naito and Yoshikawa, 2005, Quiding-Järbrink et al., 2001) and tuberculosis. Although we did not directly investigate a link between enteropathy and MMPs in this study, this finding suggests a mechanism by which *M. avium*, through induction of MMP-1 (Biancheri et al., 2013, Giuffrida et al., 2014, Steck et al., 2012), could at least be partially responsible for some of the morphological changes seen in enteropathy. MMP-1 has previously been shown to drive the immunopathological process in *Mycobacterium tuberculosis* infection in the lung (Elkington et al., 2011). Lamina propria mononuclear cells in the duodenum have been shown to express multiple MMPs after cytokine stimulation (Ciccocioppo et al., 2005).

Clearly, the use of tissue explants in vitro does not fully represent the real impact of mycobacterial pathogen-associated molecular patterns (PAMPs) on the mucosal immune response in vivo. There are several limitations to this experimental system – the tissue is traumatised during biopsy, the range of mycobacterial molecules used for stimulation is limited, the time course of the experiment is limited and fixed, and we were unable to perform a full range of dose ranging experiments as the amount of tissue available was limited. Nevertheless, the tissue is human intestinal tissue and the mucosa being stimulated has the full complement of epithelial, stromal and immune cells. Each of the tissues from the same individual were subjected to different stimuli, namely nothing (as a negative control), SEB and *S. typhimurium* LPS (as a positive controls) and *M. avium* as the main experimental stimuli. This implies that any differences in the secretion of cytokines between the negative control and *M. avium* stimulated tissues would be solely due to the *M. avium*. Furthermore, the results of the cytotoxicity assay done on the intestinal samples were within normal range. It is therefore reasonable to assume that the secretion of the different cytokines seen in this study was due to the stimulation by *M. avium*.

There is already existing evidence suggesting that environmental enteropathy is virtually ubiquitous in communities of low socio-economic status in Africa (Kelly et al., 2004, Lindenbaum et al., 1972, Prendergast and Kelly, 2012), so we postulated that non-tuberculous mycobacteria, which are also virtually ubiquitous, could contribute to intestinal mucosal re-modelling. *M. avium* is just one of the NTMs which could be implicated, and further work is required to ascertain if the effects we report here might be observed with other species.

We were unable to demonstrate the effect of *M. avium* on the secretion of MMPs 2, 8 or 9 in duodenal tissue, though it is possible that MMP-8 might have been significant in a larger study. It has been shown that MMP-1, -3 and -9 are involved in the pathogenesis of gluten sensitive enteropathy through tissue remodelling (Mohamed et al., 2006). In the lungs, it is well known that MMP-1 and MMP-9 are involved in the pathogenesis of TB. For MMP-9, this occurs through its effect on recruitment of macrophages and role in the formation of granulomas (Taylor et al., 2006) in addition to its effect on extracellular matrix. *Mycobacterium avium paratuberculosis* has been shown to enhance the expression MMP-2, -9, -13, -14, and Tissue Inhibitor of Metalloproteinase -1 (TIMP-1), in addition to IL1β and TNF-α in a murine model (Roderfeld et al., 2012).

We were also unable to demonstrate the production of IL-10 or IL-4 in duodenal samples, although we showed significant amounts of both cytokines in blood (Table 5). It is generally accepted that gut microbiota provide continuous antigenic stimulation that leads to activation of T-cells leading to intestinal injury (Sartor and Mazmanian, 2012), Gut microbiota have been implicated in the induction of regulatory B cells in the spleen and mesenteric lymph nodes through the production of IL-1β and IL-6. This inflammatory response in turn leads to the production of the anti-inflammatory IL-10 (Rosser et al., 2014).

We found evidence that *M. avium* induced the secretion of IL-1β in intestinal tissue. IL-1 is a pro-inflammatory cytokine that is activated via a variety of microbial and non-microbial mechanisms including by *Mycobacterium avium* (Elkington et al., 2011, Weber et al., 2010). IL-1β is known to be a potent stimulator of the extracellular tissue to produce MMPs including MMP-1, leading to tissue re-modelling (Dinarello et al., 2012, Garlanda et al., 2013). It has been postulated that the tissue destruction seen in environmental enteropathy could be as a result of constant activation of T-cells caused by the presence of intestinal pathogens in the lumen (Korpe and Petri, 2012, Prendergast and Kelly, 2012).

This study showed that *M. avium*-stimulated duodenal tissue secreted IL-6 more than unstimulated controls. IL-6 is a potent pleotropic inflammatory cytokine known to be involved in epithelial proliferation and wound repair (Kuhn et al., 2014), and has been shown to induce the secretion of MMP-1, -2 and -9 (Kothari et al., 2014, Sengupta and MacDonald, 2007). It has also been found to be involved in the pathogenesis of inflammatory bowel disease (Wang et al., 2003). In the present study, there was a strong correlation (*ρ* = 0.70) between IL-6 and MMP-1, suggesting that this could be one mechanism through which NTMs could lead to tissue re-modelling in enteropathy.

In conclusion, we found that *M. avium* induced the secretion of MMP-1 in duodenal tissue and in peripheral blood. *M. avium* also induced the secretion of a restricted set of cytokines in duodenal tissue, namely IL-1β and IL-6 as well as eliciting a Th1 and Th2 response in the blood. We speculate that the induction of these cytokines by *M. avium* suggests a possible pathway through which NTMs, and *M. avium* in particular, could remodel the mucosa and lead to environmental enteropathy. The induction of MMP-1 and cytokines by *M. avium* in small intestinal tissue from a tropical population suggests that environmental mycobacteria could contribute to the epithelial disruption seen in environmental enteropathy in exposed populations. Further work will be required to demonstrate if MMP-mediated mucosal re-modelling actually operates in vivo.

## Competing interests

No competing interests declared.

## Funding

Financial support for this study was provided by the Research Support Centre at the University of Zambia, School of Medicine (UNZA-SOM), through the Southern African Consortium for Research Excellence (SACORE), which is part of the African Institutions Initiative Grant (Grant code 087537) of the Wellcome Trust (Company Number 2711000), a charity (No. 210183) registered in England. Neither Wellcome Trust nor SACORE had a role in the design, conduct and interpretation of the study.

## Authors’ contributions

GC, CM and PK took part in the planning of the study, data collection, analysis and writing of the manuscript. FD took part in the planning of the study, analysis and writing of the manuscript. ES, VK, JBN took part in data collection, analysis and writing of the manuscript. All authors read and approved the final manuscript.

## Availability of data and materials

The datasets analyzed during the current study are available from the corresponding author on reasonable request.

## Consent statement

The study was approved by the University of Zambia Biomedical Research Ethics Committee. Written informed consent was obtained from all patients who took part in the study.
